# phyC: Clustering cancer evolutionary trees

**DOI:** 10.1371/journal.pcbi.1005509

**Published:** 2017-05-01

**Authors:** Yusuke Matsui, Atsushi Niida, Ryutaro Uchi, Koshi Mimori, Satoru Miyano, Teppei Shimamura

**Affiliations:** 1 Division of Systems Biology, Nagoya University Graduate School of Medicine, Nagoya, Japan; 2 Division of Health Medical Computational Science, Health Intelligence Center, The Institute of Medical Science, The University of Tokyo, Tokyo, Japan; 3 Department of Otorhinolaryngology, Graduate School of Medical Sciences, Kyushu University, Fukuoka, Japan; 4 Department of Surgery, Kyushu University Beppu Hospital, Beppu, Japan; 5 Laboratory of DNA Information Analysis, Human Genome Center, Institute of Medical Science, The University of Tokyo, Tokyo, Japan; University of Cambridge, UNITED KINGDOM

## Abstract

Multi-regional sequencing provides new opportunities to investigate genetic heterogeneity within or between common tumors from an evolutionary perspective. Several state-of-the-art methods have been proposed for reconstructing cancer evolutionary trees based on multi-regional sequencing data to develop models of cancer evolution. However, there have been few studies on comparisons of a set of cancer evolutionary trees. We propose a clustering method (phyC) for cancer evolutionary trees, in which sub-groups of the trees are identified based on topology and edge length attributes. For interpretation, we also propose a method for evaluating the sub-clonal diversity of trees in the clusters, which provides insight into the acceleration of sub-clonal expansion. Simulation showed that the proposed method can detect true clusters with sufficient accuracy. Application of the method to actual multi-regional sequencing data of clear cell renal carcinoma and non-small cell lung cancer allowed for the detection of clusters related to cancer type or phenotype. phyC is implemented with R(≥3.2.2) and is available from https://github.com/ymatts/phyC.

## Author summary

Elucidating the differences between cancer evolutionary patterns among patients is valuable in personalized medicine, since therapeutic response mostly depends on cancer evolution process. Recently, computational methods have been extensively studied to reconstruct a cancer evolutionary pattern within a patient, which is visualized as a so-called “cancer evolutionary tree” constructed from multi-regional sequencing data. However, there have been few studies on comparisons of a set of cancer evolutionary trees to better understand the relationship between a set of cancer evolutionary patterns and patient phenotypes. Given a set of tree objects for multiple patients, we propose an unsupervised learning approach to identify subgroups of patients through clustering the respective cancer evolutionary trees. Using this approach, we effectively identified the patterns of different evolutionary modes in a simulation analysis, and also successfully detected the phenotype-related and cancer type-related subgroups to characterize tree structures within subgroups using actual datasets. We believe that the value and impact of our work will grow as more and more datasets for the cancer evolution of patients become available.

## Introduction

Cancer is a heterogeneous disease. The high genetic diversity is driven by several evolutionary processes such as somatic mutation, genetic drift, migration, and natural selection. The clonal theory of cancer [1] is based on Darwinian models of natural selection in which genetically unstable cells acquire a somatic single nucleotide variant (SSNV), and selective pressure results in tumors with a biological fitness advantage for survival.

The development of multi-regional sequencing techniques has provided new perspectives of genetic heterogeneity within or between common tumors [2–6]. The read counts from multi-region tumor and matched normal tissue sequences from each patient are then used to infer the tumor composition and evolutionary structure from variant allele frequencies (VAFs); *i*.*e*., the proportion of reads containing the variant allele. Using the VAF, the cancer evolutionary histories can be reconstructed as a tree, termed a cancer evolutionary tree, which reflects the accumulation patterns of the identified SSNVs for each patient.

**Fig 1:**
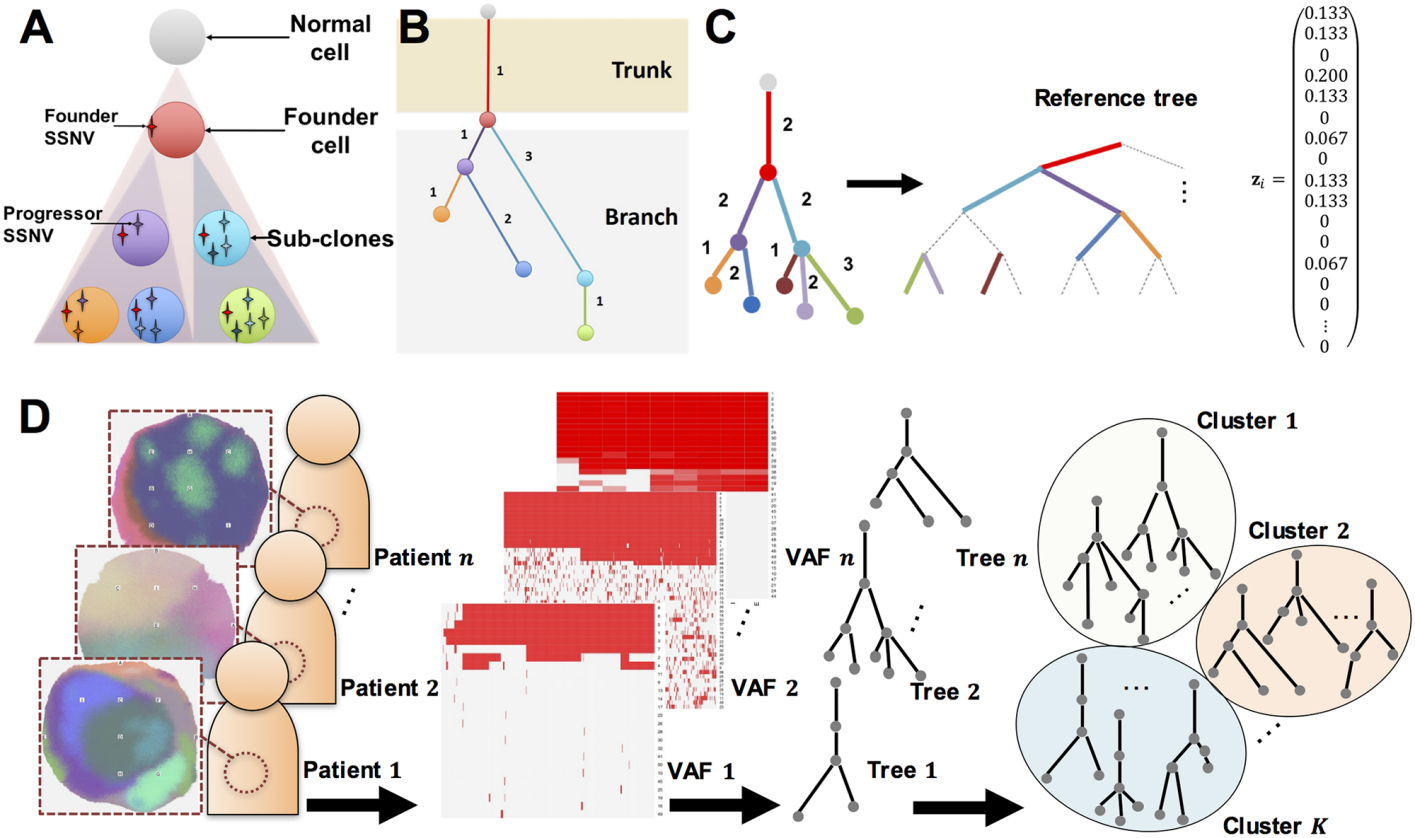
Overview of the proposed method. (A) Example of cancer evolution. A founder cell is established after a normal cell acquires several passenger mutations and driver mutations (founder SSNVs), and sub-clones evolve by acquiring progressor SSNVs. Each color (purple, orange, dark blue, light blue, and green) of circles represents different sub-clones. (B) Example of a cancer evolutionary tree in the case of (A). A root and its immediate node represent the normal cell and founder cell, respectively. Subsequent nodes indicate sub-clones and edge lengths indicate the number of SSNVs acquired in the sub-clones. (C) Example of the registration of a tree. To resolve (p1)–(p4) for comparison of the evolutionary trees, a sufficiently large bifurcated tree is constructed, which is the reference tree (note that we have omitted bifurcation from the root for clearer visualization). The tree topologies and attributes are mapped to the reference tree beginning with those with the largest depths to those with the smallest depths. In the case of a tie, the sub-trees are mapped from those with the largest edge lengths. Zero-length edges are regarded as degenerated edges (dashed lines). Edge lengths are normalized by the sum of all edge lengths within tumors. The resulting trees can be represented as edge length vectors **z**_*i*_. (D) Clustering cancer evolutionary trees to summarize the evolutionary history of cancer for each patient. The trees are reconstructed based on the VAFs and then *n* cancer sub-clonal evolutionary trees are divided into *K* subgroups based on tree topologies and edge attributes. Through the registration, *n* evolutionary trees can be represented as *m*-dimensional *n* vectors in Euclidean space, and a standard clustering algorithm can be applied.

A variety of cancer evolutionary trees can be considered as the consequence of the evolutionary principle for the underlying tumor, which may lead to resistance to chemotherapeutics and targeted therapies [19, 20]. Therefore, characterizing inter-tumor heterogeneity according to the patterns of evolutionary trees is an important strategy for developing new targeted therapies and for preventing the emergence of drug resistance. There are currently two types of cancer evolutionary trees: sample tree and sub-clonal tree. A sample tree regards each multi-region sample as being equivalent to a species in a classical tree of taxonomic phylogenetic relationships, and infers the evolutionary trees from the binary VAF profiles using classical phylogenetic algorithms such as the maximum parsimony method. A sub-clonal tree clusters SSNVs into sets of mutations with common frequency and reconstructs the lineage based on the following two assumptions [7–16]: (i) a mutation cannot recur during the course of cancer evolution, and (ii) no mutation can be lost [17] (Fig 1A). In these trees, the root and its subsequent node represent a normal cell and a founder cell, respectively. Descendant sub-clones are represented as nodes below the founder cell, and edge lengths indicate the number of SSNVs that are newly accumulated in the descendant nodes (Fig 1B). For reviews, see [18].

Several studies have suggested specific evolutionary patterns of tumors with various, and at times conflicting, results. For example, Gerlinger *et al*. [21] identified the parallel evolution of sub-clones in clear cell renal cell carcinomas (ccRCCs), whereas no such parallel evolution was evident in studies on non-small cell lung cancer (NSCLC) [22, 23]. Zhang *et al*. [23] also showed that in a relapsed group of patients, the fraction of SSNVs in sub-clones was significantly larger than that of founder cells. These studies indicate that both the branching patterns and fraction of SSNVs in sub-clones are important factors for identifying the cancer type or phenotype of related subgroups.

Although the reconstruction methods developed thus far have revealed intra-tumor heterogeneity by reconstructing individual evolutionary trees, there are currently no standardized tree comparison methods for obtaining a detailed understanding of inter-tumor heterogeneity according to evolutionary patterns with a set of reconstructed trees. Comparison of phylogenetic trees has long been discussed in the context of the evolution of species, and several comparative analytical methods have been developed, including nearest-neighbor interchanging [25], subtree transfer distance [26], quartet distance [27], Robinson-Foulds distance [28], path length metrics [29], branch length scores [30], tree edit distance [31–33], and Billera-Holmes-Vogtmann (BHV) distance [34]. However, these distances are defined for phylogenetic trees with the same set of leaves, and therefore cannot accurately deal with the following problems, (p1)–(p4), that are specific to the context of cancer evolutionary trees.

(**p1**) The parental sub-clone has one child sub-clone or more than two child sub-clones.
(**p2**) The number of sub-clones varies among patients.
(**p3**) SSNV contents differ among patients.
(**p4**) The number of detected SSNVs varies among patients.

(p1) and (p2) imply that a tree structure is not always binary, and the number of sub-clones within the tree differs among patients. Various types of new sub-clones can be produced from a common ancestral sub-clone by acquiring new sets of SSNVs, which results in complex tree structures and tree sizes. (p3) and (p4) indicate that sub-clones are rarely identical among patients since the SSNVs within a patient are quite different from those of other patients, and it is thus hard to match sub-clones between patients. In addition, the total number of SSNVs can vary substantially among patients. Therefore, according to experimental conditions, it is important to adjust for these effects to effectively compare the trees. These problems motivated us to develop a method for the effective comparison among tree via transformation of the tree topologies and edge attributes, a procedure we refer to as tree registration.

In this paper, we propose a new clustering method for cancer evolutionary trees based on tree topologies and edge attributes that describe the relationships of sub-clones and the number of SSNVs that accumulate in the sub-clones. Our conceptual framework is based on object-oriented data analysis [24], in which the observation units are non-numeric objects such as functions and trees. The main contributions of this paper are development of (i) a tree registration method for cancer evolutionary trees, (ii) a clustering method of the registered trees, and (iii) an evaluation method of the clusters, which can be applied using our software phyC in the R environment.

In the registration, we resolve the issues raised in (p1)–(p4) through development of a method for transforming tree objects by mapping tree topologies and their attributes to make the trees comparable (Fig 1C). The registered trees are embedded in Euclidean space, which enables defining the distance between the cancer evolutionary trees. Based on this distance, we divide a set of the trees into several sub-groups with a clustering method (Fig 1D). We developed two tools for interpretation of the clusters: multidimensional scaling (MDS) and a sub-clonal diversity plot.

We evaluated the performance of phyC using simulated data that mimic the actual scenarios. We also demonstrate the applicability of phyC using two actual datasets from patients with ccRCCs [21] and NSCLC [23], respectively, to show the interpretability of the clustering results. phyC is implemented with R(≥3.2.2) and is available from https://github.com/ymatts/phyC.

## Methods

We denote *n* reconstructed cancer evolutionary trees as *X* = {*x*_*i*_; *i* = 1, 2, …, *n*}, and the edges and edge lengths are denoted as {*e*_*ij*_; *i* = 1, 2, …, *n*, *j* = 1, 2, …, *m*_*i*_} and {|*e*_*ij*_|; *i* = 1, 2, …, *n* *j* = 1, 2, …, *m*_*i*_}, respectively. Without loss of generality, {*e*_*i*1_; *i* = 1, 2, …, *n*} indicates the edge from the normal cell to the founder cell. Given the number of terminal nodes *N*_*i*_; *i* = 1, 2, …, *m*_*i*_, we set depth (*i*.*e*., the number of edges in the path from the root to the terminal node) as *d*_*ik*_(*i* = 1, 2, …, *n*; *k* = 1, 2, …, *N*_*i*_).

### Registration

We developed a registration method for the cancer evolutionary trees. The goal of the registration is to transform the observed trees such that dissimilarities can be defined with consideration of the tree topologies and edge attributes. To solve the problems (p1)–(p4), we provide the following approaches, (q1) and (q2):

(**q1**) Reference tree encoding
(**q2**) Normalizing edge lengths

To account for different tree structures and sizes as raised in (p1), we consider a reference tree-encoding approach that is similar to [35]. In this approach, we prepare a very large bifurcated tree called a reference tree (corresponding to the maximum tree in [36] and encode the observed tree topologies and edge lengths onto the reference. Zero-length edges are regarded as degenerated edges (Fig 1C). The advantage of this approach is that once we encode the observed tree onto a reference tree, the comparison can be simply achieved for trees of the same structures and sizes. To account for the issue (p4), we developed a method for normalization of the edge length to remove the bias in the detected number of SSNVs.

Here, we describe the details of the registration method. First, we set the maximum depth in *X* as *d*_*max*_(*X*) = max {*d*_*ik*_; *i* = 1, 2, …, *n*, *k* = 1, 2, …, *N*_*i*_} and define the reference tree as follows.

**Definition 1** (**Reference tree**) *The reference tree is a bifurcated tree with the minimum depth of d*_*max*_(*X*).

Thus, the reference tree has *m* = 2(2^*d*_*max*_(*X*)^ − 1) edges (Fig 1C). We denote the reference tree as *X*_*ref*_ with edges and edge lengths *E*_*k*_; *k* = 1, 2, …, *m* and |*E*_*k*_|; *k* = 1, 2, …, *m*, respectively. The registration can then be defined with the reference tree.

**Definition 2** (**Registration**) *Registration is a mapping f*: *X* ↦ *X*_*ref*_.

We define the mapped trees as *Y* = {*f*(*x*_*i*_); *i* = 1, 2, …, *n*}, and more specifically, the mapped edge and edge length are set to {*E*_*ik*_; *i* = 1, 2, …, *n*, *k* = 1, 2, …, *m*} and {|*E*_*ik*_|; *i* = 1, 2, …, *n*, *k* = 1, 2, …, *m*}, respectively. The number of edges differs between the observed tree and the reference tree, and we also need to account for any unmapped edges. Since the degenerated edges can be regarded as the zero-length edge when considering the distance of trees [36], we can define |*E*_*ik*′_| = |*e*_*ij*_|; *k*′ ∈ *A* for the mapped edge index set *A* ⊆ {1, 2, …, *m*}, and define |*E*_*ik*′_| = 0; *k*′ ∈ *B* for the unmapped edge index *B* = {1, 2, …, *m*}\*A*.

To resolve (p3), we developed the mapping rule *e*_*ij*_ ↦ *E*_*ik*_ for *j* = 1, 2, …, *m*_*i*_, *k* = 1, 2, …, *m*, such that the observed trees are mapped onto the reference tree beginning with sub-trees with the largest depths and moving on to those with the smallest depths (Fig 1C). When the depths are the same among the sub-trees, we use the edge length and map the sub-trees beginning with those with the largest edge lengths (Fig G in S1 Text).

In the last step of the registration, we perform normalization for the edge length. Zhang *et al*. [23] importantly suggested in NSCLC study that patients with relapsed disease had larger fractions in their primary tumors (average 41% in patients with relapse versus 24% in patients without relapse, p = 0.045 by t test). Therefore, we consider that the ratio of the number of accumulated SSNVs is an important factor to characterize and compare the cancer evolutionary trees, and we divided each edge length by the total number of SSNVs within patients.

$$\begin{array}{c} \left|E_{ik}\right|/\sum_{k=1}^{m}\left|E_{ik}\right|,\;\text{for}\;\text{all}\;k. \end{array}$$ (1)

### Clustering set of registered trees

To define the dissimilarity between the registered trees, we begin with the space of the set of the registered trees. Billera *et al*. [34] proposed the concept of a continuous tree space-associated geodesic distance metric as a natural way to embed and compare phylogenetic trees. This tree space consists of a set of Euclidean regions, called orthants, one for each tree topology. Orthants are joined together whenever one tree topology can be made into another by exchanging edges between the trees. Within an orthant, the coordinates of each point represent the edge lengths for a particular tree with the topology associated with that orthant. Since we only encode the observed trees onto the reference tree with the same topology, the registered trees do indeed lie in the same orthant as a special case of BHV space.

**Corollary 1** (**Euclidean embedding**) *The registered trees lie in Euclidean space*.

We represent the registered tree as a vector, whose elements correspond to each edge length as $z_{i}^{\prime }=\left(z_{i1},z_{i2},\ldots ,z_{im}\right);z_{ij}\in \text{R}$. Note that zero length edges are regarded as degenerated edges. Thus, *n* registered trees are represented as the *n* × *m* matrix $Z^{\prime }=\left(z_{1},z_{2},\ldots ,z_{n}\right)$.

We define the dissimilarity as follows:

$$\begin{array}{c} s\left(x_{i},x_{j}\right)≔\sqrt{{\left(z_{i}-z_{j}\right)}^{\prime }\left(z_{i}-z_{j}\right)}. \end{array}$$ (2)

The basic statistics of the cancer evolutionary trees can also be defined. The tree average is defined as $\mu =\frac{1}{n}\sum_{i=1}^{n}z_{i}$ and the tree variance is defined as $\sigma^{2}=\frac{1}{n}\sum_{i=1}^{n}{\left(z_{i}-\mu \right)}^{\prime }\left(z_{i}-\mu \right)$.

Based on the tree representation with **Z**, which can be regarded as *n* observations with an *m* features matrix, we can simply apply standard clustering algorithms and divide the *n* trees into subgroups. Hierarchical clustering was then implemented using phyC. To determine the number of clusters automatically, we applied the gap statistics criterion [37] with the NbClust R package [38].

### Graphical representation

Interpreting clustering results is a key issue for tree comparison, which requires understanding the features of the cancer evolutionary trees in clusters. In particular, visual representation can be a powerful tool for such interpretation. Therefore, we developed two computational tools for comparing trees and understanding the cluster features.

#### MDS

To effectively compare the trees, we approximately embedded the registered trees into lower-dimensional Euclidean space. For this purpose, we applied classical MDS (CMDS) [39], which is a dimension-reduction technique based on singular value decomposition. We will here omit the details of the CMDS algorithm and briefly describe the method below. Given the symmetric distance matrix *S* = {*s*_*ij*_; *i*, *j* = 1, 2, …, *n*}, the double-centered matrix

$$\begin{array}{c} B=-\frac{1}{2}HS^{2}H \end{array}$$ (3)

is positive semi-definite, where $H=1-\frac{1}{n}1_{n}{1_{n}}^{\prime }$, and can be diagonalized as

$$\begin{array}{c} B=U\Lambda U^{\prime }. \end{array}$$ (4)

The constructed coordinates are obtained by $X=U\Lambda^{\frac{1}{2}}$. For this purpose, we use the distance that is defined in Eq (2). CMDS requires knowing the number of dimensions, which we set to two for the purpose of convenient visualization. In phyC, we overlaid the tree shapes over the coordinates and visually compared the tree structures based on dissimilarity.

#### Sub-clonal diversity plot

To visualize how sub-clones evolve with respect to SSNV accumulation, we apply the concept of a lineage-through-time (LTT) plot, which is commonly used for visualizing the timing of speciation events in studies of the birth-death process. The LTT plot generally describes the time vs. number of lineages; in the present case, this is expressed as the number of sub-clones (y-axis) vs. the fraction of accumulated SSNVs (x-axis) and the plot is referred to as a sub-clonal diversity plot. This plot represents the growth rate of the number of sub-clones when a certain percentage of mutations accumulate. In the plot, *y* = 1 means that there is no sub-clone, and thus only a normal cell exists, and *y* = 2 indicates that there is a founder cell. For example, (*x*, *y*) = (0.3, 2) indicates that the founder cell is established with the accumulation of 30% SSNVs. For *y* > 1, the growth curve in the plot represents how many sub-clones emerged for a given fraction of SSNVs. If the curve is upright, the sub-clones evolve with a small fraction of SSNV accumulation, and conversely, if the curve grows with gradual steps, the sub-clones acquire a relatively large fraction of SSNVs. A gradual growth curve was observed in the case of parallel evolution shown in [21], which will be demonstrated below in the implementation of the ccRCC dataset.

## Results

### Simulation analysis

We evaluated the performance of the proposed method using simulation data that reflects real situations. The main purpose of simulation is to show the effectiveness of the registration process for cancer evolutionary tree classification when compared to methods without the registration. Moreover, we investigated whether the differences in tree topology or edge length are more important when comparing tree objects.

There are two types of tree comparison methods: one is based on only tree topologies, and the other is based on both tree topologies and edge attributes. We examined phyC from the viewpoint of classification performance for tree topologies, edge length, and both. We conducted the following three simulations:

**Simulation I** Comparison of tree topologies
**Simulation II** Comparison of the edge lengths of tree
**Simulation III** Comparison of both tree topologies and edge lengths

Simulation I was conducted to examine whether phyC can classify tree topologies, *i*.*e*., the edge lengths are all the same. In simulation II, we examined the classification performance of phyC for edge length differences. Simulation III was designed to examine the performance of phyC for both tree topology and edge length differences between tree objects.

To evaluate the clustering results, we adopted three external clustering validation indices [40], which are described in S1 Text: purity (PR), normalized mutual information (NMI), and Rand index (RI). In the following three simulations, we created 100 replicates of each dataset and evaluated the mean and standard deviation of PR, NMI, and RI.

#### Simulation I

To simulate cancer evolutionary tree topologies, we manually created four classes of tree topologies used in Yuan *et al*. [41]: monoclonal (MC), polyclonal-low (PL), polyclonal-high (PH), and mutator phenotype (MT). Based on the four classes of tree topologies, we generated random topologies via tree editing with various dispersion parameters described in S1 Text. We obtained 10 trees for each class, and simulations were conducted for various dispersion parameters. The results were compared to those of the tree edit distance (TED) using RTED [33] and to those of the shortest path distance (SPD).

#### Simulation II

**Fig 2:**
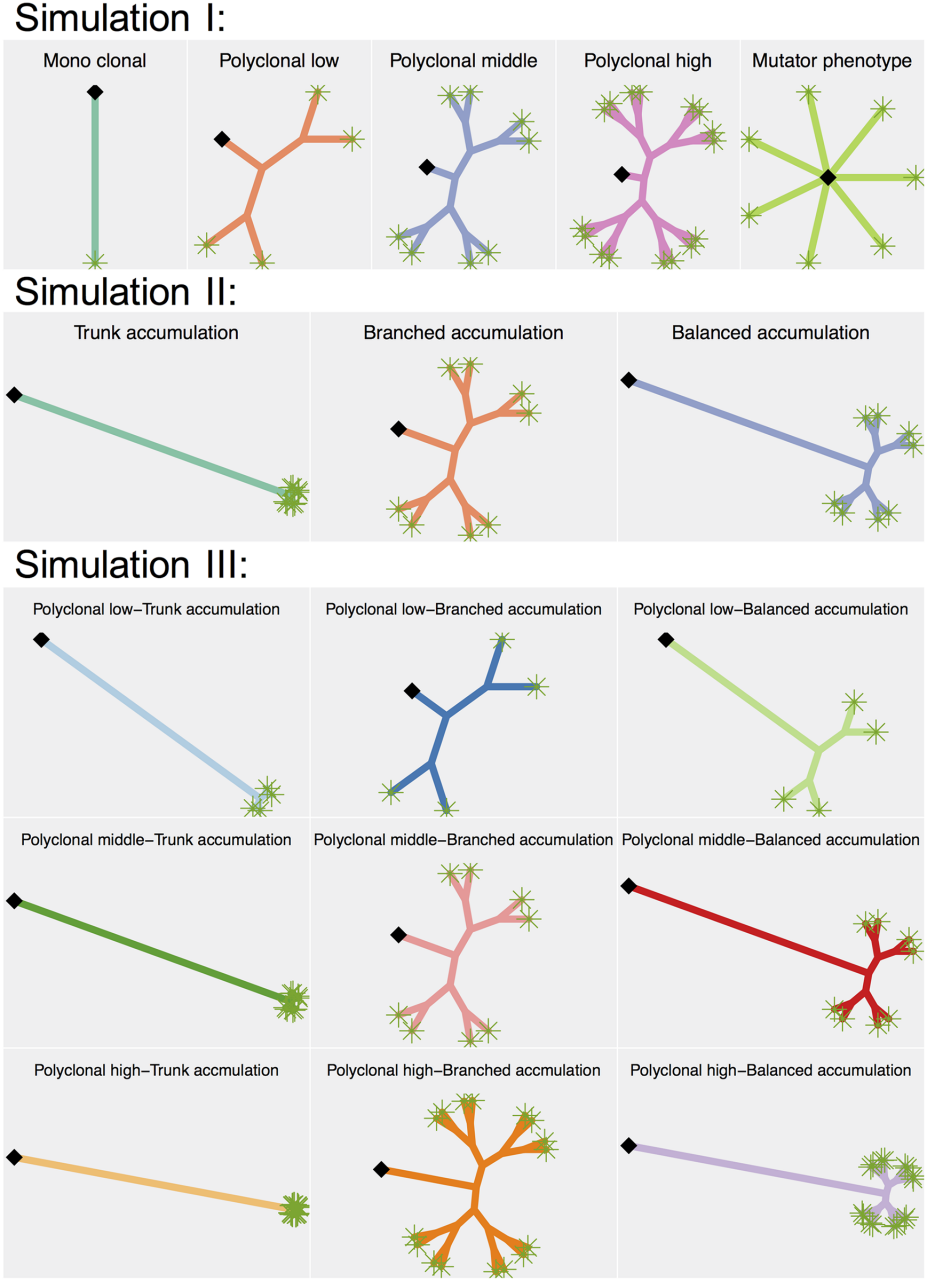
Classes of cancer evolutionary trees in the simulations. **Simulation I**: Five classes of tree topologies were considered: monoclonal (MC), polyclonal-low (PL), polyclonal-middle (PM), polyclonal-high (PH), and mutator-phenotype (MT). **Simulation II**: Three classes of edge lengths of the tree are considered: trunk accumulation (TR), branched accumulation (BR), and balanced accumulation (BL). **Simulation III**: Nine classes of trees are considered: polyclonal-low trunk accumulation (PL-TR), polyclonal-low balanced accumulation (PL-BL), polyclonal-low branch accumulation (PL-BR), polyclonal-middle trunk-accumulation (PM-TR), polyclonal-middle balanced-accumulation (PM-BL), polyclonal-middle branch-accumulation (PM-BR), polyclonal-high trunk accumulation (PH-TR), polyclonal-high balanced accumulation (PH-BL), and polyclonal-high branch accumulation (PH-BR)

Zhang et al. [23] showed that the ratio of the number of SSNVs in the trunk to branches was significantly different between the recurrent and non-recurrent group, suggesting that the ratio of the edge length of the trunk to that of the branches can be related to phenotype. We considered three classes of SSNVs accumulation patterns with regard to the edge length of cancer evolutionary trees: trunk-accumulation (TR), balanced-accumulation (BL), and branch accumulation (BR), as shown in Fig 2. In TR and BR, most of the SSNVs are accumulated in the trunk and branch, respectively. In BL, the SSNVs are equally distributed in the trunk and branch. Based on these three classes, we generated the random trees described in S1 Text. We obtained 10 trees for each class, and simulations were conducted for different variances. The results were compared to those of the branch length score (BScore).

#### Simulation III

We created nine classes of tree objects considering both tree topology and edge length (Fig 2). We used the three tree topologies from simulation I, PL, PM and PH, and adopted the three classes of SSNVs accumulation patterns from simulation II, TR, BL, and BR, to each tree topology. We obtained nine classes that represent a combination of cases in simulations I and II, named polyclonal-low trunk-accumulation (PL-TR), polyclonal-low balanced-accumulation (PL-BL), polyclonal-low branch-accumulation (PL-BR), polyclonal-middle trunk-accumulation (PM-TR), polyclonal-middle balanced-accumulation (PM-BL), polyclonal-middle branch-accumulation (PM-BR), polyclonal-high trunk-accumulation (PH-TR), polyclonal-high balanced-accumulation (PH-BL), and polyclonal-high branch-accumulation (PH-BR). Based on these nine classes, we generated the random trees described in S1 Text. We obtained 10 trees for each class, and simulations were conducted for different variances. The results were compared to those of the BScore.

#### Simulation results

**Fig 3:**
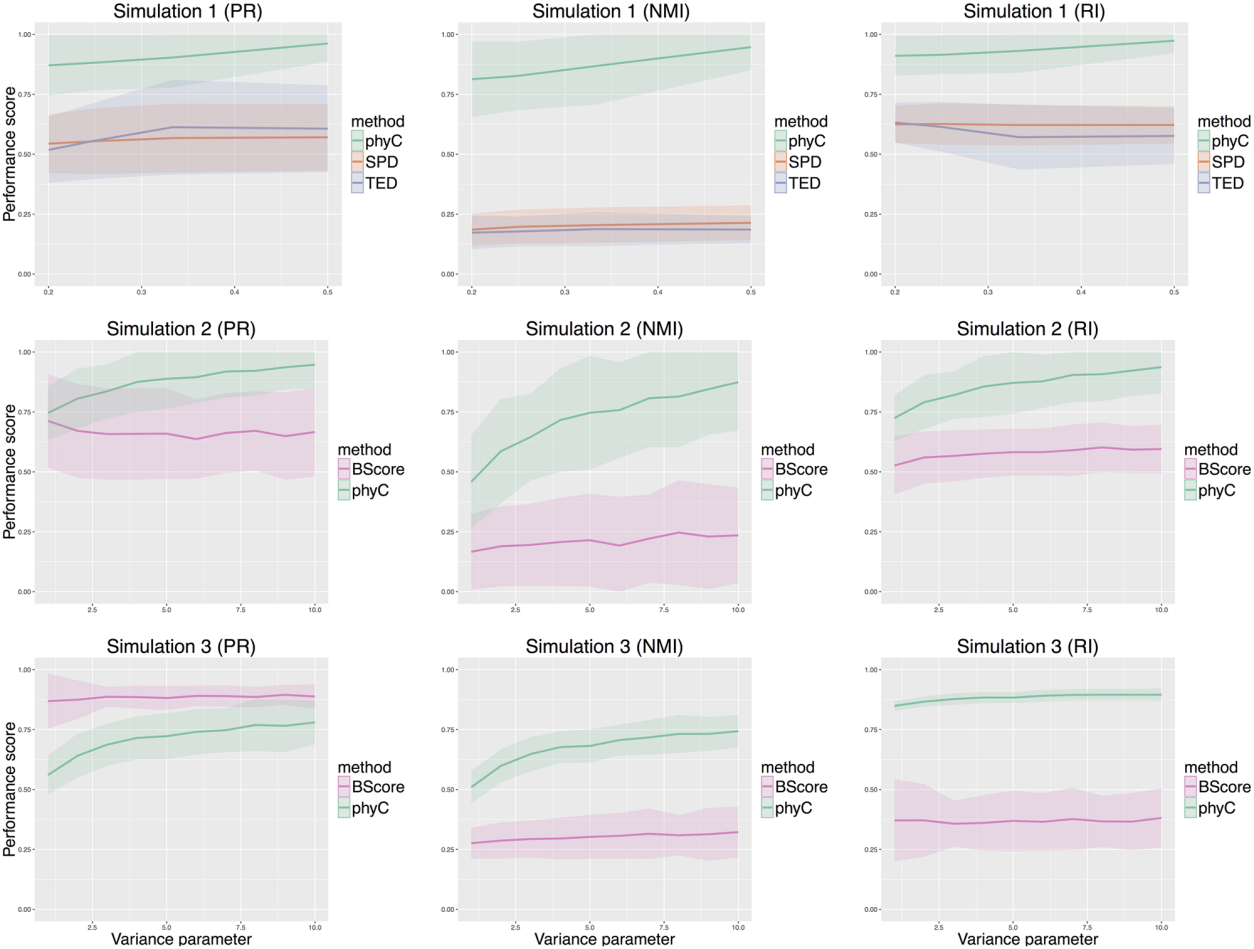
Results of the simulations. Each row of panels represents the simulation type (simulation I, II, and III), and each column represents the external clustering validation indices: purity (PR), normalized mutual information (NMI), and Rand index (RI). The horizontal axis of each graph is the variance parameter defined in S1 Text, and the vertical axis is the external validation index. The bold lines and the bands indicate the mean and 95% confidence interval of the index for 100 replicates of each dataset.

Fig 3 shows the results of each simulation, where the x-axis and y-axis represent the variance index and performance score, respectively. The variance index corresponds to the descending order of the variance, and the performance scores are defined by external clustering validation indices.

In simulation I, phyC outperformed the other methods without the registration, although the performance decreased with an increase in the variance. The performance of NMI of phyC was much higher than that of the other methods, in contrast to those of PR and RI, which indicated that the clusters of TED and SPD included more objects from the other classes than that of phyC. The good performance of phyC is attributed to the mapping for tree topologies in the registration, which allows us to focus only on the tree shapes without considering node labels in contrast to the other methods. The mapping process is clearly effective when considering the differences of cancer evolutionary tree topologies.

In simulation II, phyC showed higher clustering performance than BScore, without the registration. When the variance was large, the performances of phyC and BScore were close; however, the performance of phyC increased with smaller variances. Similar to the results of simulation I, the performance of NMI of phyC was much higher than that of the other methods. These results are attributed to the mapping and edge length normalization in the registration, which allows us to focus on the edge length ratio with regard to SSNVs accumulation patterns.

In simulation III, the performance score of BScore with PR was higher than that of phyC, but the scores for NMI and RI showed the opposite results. In BScore, most of the objects are assigned to a few large clusters, and the remaining fractions of objects are distributed to several small clusters, which results in a high PR score. The good performance of phyC is attributed to the mapping and edge length normalization in the registration, as in simulations I and II. The performance of phyC tended to become saturated at around 0.75 and 0.90 in NMI and RI, respectively, compared to the performance of simulation I and II. This is because the three classes defined by PL-TR, PM-TR, and PH-TR were assigned to the same clusters, which indicates that phyC tends to emphasize the difference in edge length rather than the difference in tree topology when the differences of edge length are extremely large.

These results indicate that our method is effective for capturing the differences in the shapes of cancer evolutionary trees.

### Real data

We here demonstrate the application of our proposed method using an actual ccRCC dataset [21] and an NSCLC dataset [23], consisting of 8 and 11 multi-regional tumor samples with VAFs collected among 587 and 7,026 SSNVs, respectively. Since both studies used the maximum parsimony method to reconstruct the cancer evolutionary trees, we also adopted this method to analyze the datasets with our approach. We binarized the VAF profiles with *VAF* ≥ 0.05 as one and otherwise zero. Using the binary profile, we estimated the phylogenetic trees using the function acctran in the R package phangorn [42] and we obtained 19 cancer evolutionary trees.

#### ccRCC dataset

**Fig 4:**
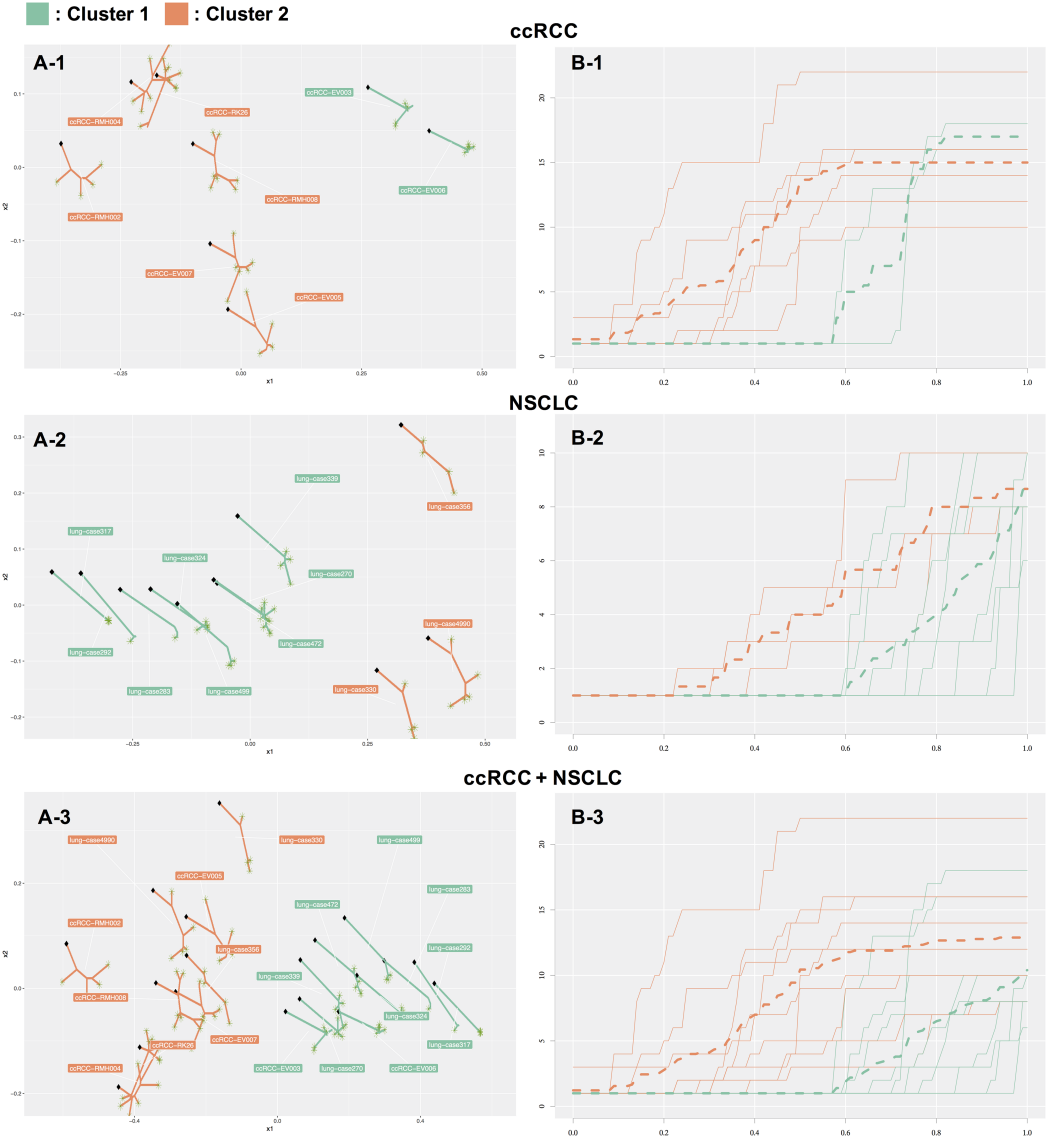
(A-1) Three clusters of the ccRCC dataset. The x-axis and y-axis are the lower dimensions reconstructed by CMDS. Clusters 1 (green) and 2 (orange) reflect drug-sensitive evolution and parallel evolution, respectively; we cannot provide a valid interpretation for cluster 3 (purple) at present. (B-1) Sub-clonal diversity plot of the ccRCC dataset. The x-axis and y-axis are the fraction of accumulated SSNVs and the number of sub-clones, respectively. Each color corresponds to the clusters shown in (A). Expansions in cluster 1 occurred with *x* = 0.6; (*i*.*e*., the proportion of SSNVs in the trunk is 60%). This result is in contrast to that obtained for cluster 2 (*x* = 0.2) and cluster 3 (*x* = 0.1). Trees in cluster 2 show gradual growth of sub-clonal diversity curves, indicating that these sub-clones acquire a relatively large fraction of SSNVs. The sub-clones independently evolve in spatially distinct regions [21]. (A-2) Two clusters in the NSCLC dataset. Clusters 1 (green) and 2 (orange) reflect the non-recurrent and recurrent group, respectively. Only case 270 and case 356 were misclassified to clusters 1 and 2, respectively. (B-2) Sub-clonal diversity plot of the NSCLC dataset. Each color corresponds to the clusters shown in (A-2). (A-3) Two clusters in the ccRCC and NSCLC datasets combined. Clusters 1 (green) and 2 (orange) represent the cancer types NSCLC and ccRCC, respectively. (B-3) Sub-clonal diversity plot of the ccRCC and NSCLC datasets.

The ccRCC dataset consists of eight evolutionary trees with clinical information related to treatments. We divided the eight evolutionary trees of the ccRCC dataset into three subgroups using hierarchical clustering with Ward’s method. Table 1 shows the clustering result, and a configuration of the eight trees with CMDS and a dendrogram are shown in Fig 4A-1 and Fig A in S1 Text, respectively. We also show in Fig D of S1 Text the tree averages that are mean of edge length of registered trees in each cluster.

**Table 1:** Clustering result of the ccRCC dataset.

Cluster	Sample name
Cluster 1	EV003, EV006
Cluster 2	EV005, EV007, RMH008, RMH004, RK26, RMH002

The tree averages in Fig D in S1 Text indicate that the tree shape of cluster 1 is characterized by trunk accumulation-like shape in contrast to cluster 2 and 3 that are characterized by balanced accumulation and branched accumulation-like shapes, respectively.

Cases EV003 and EV006 in cluster 1 received pretreatment with everolimus [21]. The sub-clonal diversity plot shown in Fig 4B-1 demonstrates that sub-clonal expansions in cluster 1 occurred after 60% of the SSNVs accumulated, in contrast to cluster 2 (10%), which may indicate that the drug interrupts acquisition of SSNVs in the sub-clones, leading to lower genetic diversity of these sub-clones. Cluster 1 reflects the drug-sensitive sub-clones group, and the result corresponds to the interpretation provided by Gerlinger *et al*. [21].

Cases EV005, EV007, RMH004, RMH008, RK26, and RMH002 in cluster 2 acquired a large fraction of SSNVs in the sub-clones (Fig 4B-1). Comparison of the original tree shape demonstrated that the long branches are followed by several private branches. The samples of EV005, EV007, RMH004, and RMH008 were reported as sub-clones of parallel evolution, *i*.*e*., each sub-clone independently evolved in spatially distinct regions [21].

These findings demonstrate that our method can produce interpretable clusters for the drug-sensitive group and parallel evolution group in the ccRCC dataset.

#### NSCLC dataset

The NSCLC dataset consists of 11 evolutionary trees with the following clinical information: staging (IA, IIA, IIIA, and IB), smoking status (former, current, and never), and recurrence (yes and no). We divided the 11 trees into two subgroups using hierarchical clustering with Ward’s method. The clustering result is shown in Table 2, and the configuration of CMDS and a dendrogram are shown in Fig 4A-2 and Fig B in S1 Text, respectively. The tree averages are shown in Fig E in S1 Text.

**Table 2:** Clustering result of the NSCLC dataset.

Cluster	Sample name
Cluster 1	case 317, case 292, case 283, case 324, case 499 case 472, case 339, case 270
Cluster 2	case 330, case 4990, case 356

The tree averages in Fig E in S1 Text indicate that the tree shape of cluster 1 and 2 are characterized by trunk accumulation and balanced accumulation-like shape.

Case 330 and case 4990 in cluster 2 are labeled as the recurrent group, but case 356 is not. As shown in Fig 4B-2, there are several long horizontal regions in the sub-clonal diversity curve, which indicates that each sub-clone acquired a large portion of SSNVs. This implies that the sub-clones contained a large fraction of the SSNVs after diverging from the founder cell, *i*.*e*., representing a genetically new generation. Zhang *et al*. [23] reported a similar observation, in which they found a significant difference (t-test) in the average fractions of SSNVs between the recurrent group and non-recurrent group. Case 356 is labeled as non-recurrent; however, it shows a large fraction of SSNVs in the sub-clones compared to that of the non-recurrent group, leading to a similar tree shape to that of trees of the recurrent group.

Cluster 1 consists of non-recurrent cases, except for case 270. Fig 4B-2 shows small horizontal regions in the sub-clonal diversity curve, which indicates that each sub-clone acquired a smaller portion of SSNVs compared to that observed in the sub-clones of cluster 2; that is, most of the SSNV acquisition events had already occurred as founder mutations. Although case 270 is labeled as recurrent, it shows a lower fraction of SSNVs in the sub-clones compared to that of the recurrent group; as a result, its tree shape resembles that of trees of the non-recurrent group.

The difference between the two clusters indicates that acquisition of a large fraction of SSNVs in sub-clones may influence the survival of cancer patients, and phyC could capture this feature and correctly classify them, which is consistent with the results of Zhang et al. [23].

#### Comparison of the ccRCC and NSCLC datasets

In addition to establishing the evolutionary pattern of a certain cancer type or sub-type, it is also interesting to compare the evolutionary patterns of different types of cancer. Therefore, we compared the evolutionary trees derived from the ccRCC and NSCLC datasets. We applied phyC to the 19 trees reconstructed as described in the previous sub-sections, which were divided into two distinct clusters (Table 3, Fig 4A-3 and Fig C in S1 Text, Fig F in S1 Text).

**Table 3:** Clustering result of the ccRCC and NSCLC datasets.

Cluster	Sample name
Cluster 1	case 317, case 292, case 283, case 324, case 499 case 472, case 339, case 270, EV003, EV006
Cluster 2	EV005, EV007, RK26, RMH002, RMH004 RMH008, case 330, case 4990, case 356

The tree averages in Fig F in S1 Text indicate that the tree shape of cluster 1 and 2 are characterized by trunk accumulation and branched accumulation-like shape.

The trees could be mainly classified according to cancer type. One of the main features of the ccRCC evolutionary trees in cluster 2 was the acquisition of a large fraction of SSNVs in the sub-clones, leading to the tendency of parallel evolution. In contrast to the ccRCC trees, the NSCLC evolutionary trees showed that a large fraction of SSNVs was acquired in the trunk, and not in the sub-clones, which confirmed that the important event had already occurred in the early stage of SSNVs acquisition [23].

Some of the trees were classified with different cancer types, including case 330, case 4990, and case 356 in cluster 2, and EV003 and EV006 in cluster 1. Case 330 and case 4990 in the NSCLC dataset are part of the recurrent group, and the tree shape of case 356 is similar to that of the recurrent group (Table 3). A large fraction of SSNVs of case 330, case 4990, and case 356 accumulated in branches, as in the ccRCC samples. EV003 and EV006 in the ccRCC dataset are samples of drug-sensitive tumors, and their tree shapes resemble those of NSCLC trees, which further supports that the drug interrupts the accumulation of SSNVs in the sub-clones.

## Discussion

We developed phyC, which was designed for clustering a set of cancer evolutionary trees to characterize cancer evolutionary patterns according to tree shape, based on analysis of tree topologies and edge attributes. Using this approach, we effectively identified the evolutionary patterns with different degrees of heterogeneity in a simulation study. We also successfully detected the phenotype-related and cancer type-related subgroups when applying this method to actual ccRCC and NSCLC data.

Considering the generally high level of inter-tumor heterogeneity, it is important to be able to identify phenotype- or cancer type-related evolutionary patterns. Previous studies have classified and interpreted the branching patterns of such sub-clones with manual methods, and then separately analyzed the compositions of SSNVs in each sub-clone. However, development of a quantitative analysis method is required to deal with datasets containing a large number of patients with cancer evolutionary trees to characterize and interpret the evolutionary patterns.

Our approach relies on reconstruction methods of evolutionary trees, and we used a parsimony approach that is widely adopted in studies of multi-regional sequencing. The proposed method only requires knowledge of the edges and edge attributes of rooted trees, and is therefore widely applicable to outputs of other recently developed state-of-the-art reconstruction methods, which allowed us to consider the heterogeneous mixture of cells within a sample.

There are several limitations of the present method that are worth mentioning, which should be tackled in further investigations. First, we have ignored the specific content of SSNVs in the sub-clones. We believe that this is a reasonable assumption to some extent, since the variation of SSNVs is too large to yield an effective comparison. However, the effects and consequences of different types of SSNVs can also vary, such as driver mutations or passenger mutations. Thus, when comparing edges with the same lengths from different trees, the two edges may not actually be equivalent if driver genes are included in one edge but not in the other. The first cut distinction between driver and passenger mutations could also simplify the algorithm and improve its running time. Therefore, a method that can incorporate the effect of driver genes in the sub-clones should be explored in future work.

Second, we have here only considered the SSNVs accumulating in the evolutionary trees, ignoring potential copy number or epigenetic aberrations; however, these factors may also affect heterogeneity within a tumor. Multi-regional sequence analysis has been performed using exome sequencing as well as copy number, methylation, and mRNA expression array profiling, providing an integrated interpretation of cancer evolution [43]. To determine the evolutionary patterns from these integrated data, our method can be extended to the case of multivariate edge attributes, including copy number variations and hyper- or hypo-methylation, as well as other genetic and epigenetic aberrations.

Finally, we did not take into account the potential effects of regional sampling biases and individual variations among tumors or patients. Gerlinger *et al*. [21] pointed out that increasing the sequenced regions of samples might lead to additional detection of sub-clones, and thus the complexity of inferred evolutionary trees might be affected by the sampling strategy. Therefore, a method for sampling bias reduction is needed to improve the clustering accuracy and plausible interpretation.

Our proposed approach represents the first practical method to quantitatively and accurately compare a variety of evolutionary trees with different structures, sizes, and labels, and with biases of edge length, while further allowing for biological interpretation. Our results imply that this approach has potential applications for personalized medicine such as predicting the outcomes of chemotherapeutics and targeted therapies, *e*.*g*., drug-resistance, based on evolutionary trees. We believe that the value and impact of our work will grow as more and more multi-regional sequencing datasets of patients become available.

## Supporting information

S1 Text S1 Text contains detailed procedure of generating simulation data, external evaluation criteria for clustering, sampling effects on the clustering results, supporting figures and tables (Fig A–Fig G in S1 Text and Tables A–F in S1 Text). (PDF)

S1 File S1 File contains an R Markdown file for reproducing all the figures in this manuscript and the related data can be downloaded from https://github.com/ymatts/phyC/tree/master/misc. (HTML)
