# Supplementary material for: phyC: Clustering cancer evolutionary trees
Source: PLoS Comput Biol. 2017 May 1;13(5):e1005509. doi: 10.1371/journal.pcbi.1005509 (PMC5432190; doi:10.1371/journal.pcbi.1005509)
Supplement: S1 Text — (PDF) [file pcbi.1005509.s001.pdf]

phyC: Clustering Cancer Evolutionary Trees

Yusuke Matsui, Atsushi Niida, Ryutaro Uchi,
Koshi Mimori, Satoru Miyano, Teppei Shimamura

S-1 Details of simulations

S-1-1 Negative binomial distribution

An integer valued random K is said to follow a negative binomial distribution with parameter $p(0 < p < 1)$, $s(0 < s)$ if

$$\Pr(K = k) = \binom{k+s-1}{s-1} p^s (1-p)^k. \tag{1}$$

This two parametric distribution can alternatively parametrised in terms of mean μ and size s, called dispersion parameter, via

$$p = \frac{s}{s+\mu}. \tag{2}$$

where the variance is $\sigma^2 = \mu + \frac{\mu^2}{s}$. We denote $K \sim NB(\mu, s)$ if the random variable K follows negative binomial distribution.

S-1-2 Details of simulation I

We generated random trees via generating random tree topologies as follows. Let $E = \{e_i; i = 1, 2, \ldots, m\}$ be edges of a tree defined as any of the five types defined in Figure 2. We generated random trees via editing edges e. The editing operation, denoted by a function $OP(E, N)$ where e is edge set and N is the number of the operation per tree , consists of "add" and "delete" the edge, and "none" (does nothing) that are randomly chosen. We controlled topological variance by the number of the operations per tree denoted as N with larger one resulting in larger topological variance. We generated 10 trees for each classes of tree topology and examined various topological variances $N = 2, 3, 4, 5$. In Figure 3, the variance index corresponds to $\frac{1}{N}$.

S-1-3 Details of simulation II & III

We generated random trees via generating random edge length as follows. Let $l = (l_1, l_2, \ldots, l_m)$ be vector of edge lengths of a tree defined as any of the three types of simulation II and the nine types of simulation III in Figure 2. We generate random trees via generating random edge length independently using negative binomial distribution. We set size parameter s and the mean parameter is set for each edge length l_i as $\mu_i = l_i$ $(i = 1, 2, \ldots, m)$. We generate a random tree with edge length $(Z_1, Z_2, \ldots, Z_m)$ via $Z_i \sim NB(\mu_i, s)$. In this simulation, we generated 10 trees for each class and examined various size parameters $s = 1, 2, \ldots, 10$.

S-1-4 External clustering validation indices

Purity (PR) measures accuracy of cluster assignments to the correct classes that are defined by the majority cluster,

$$\mathrm{PR}(\Omega, \mathcal{C}) = \frac{1}{N} \sum_k \max_j |\omega_k \cap c_j|, \tag{3}$$

where $\{\omega_k;\ k = 1, 2, \ldots, n\}$ is set of clusters and $\{c_j;\ j = 1, 2, \ldots, m\}$ is set of classes. PR lies in $[0, 1]$ and higher PR is considered as good. However, since PR doesn't consider the number of clusters, high PR is easy to achieve when the number of clusters is large, *e.g.,* PR is 1 if each object gets its own cluster. PR also doesn't consider the false positive rate. The other two indices below incorporate false positive rate or the number of cluster into their evaluation index.

Rand index (RI) measures the accuracy of the clustering assignments. We consider true positive (TP), true negative (TN), false positive (FP), and false negative (FN). If a pair of similar clusters are assigned to the same cluster, the pair is counted as TP and conversely, if a pair of dissimilar clusters are assigned to the different cluster, the pair is counted as TN. The FP and FN counts the pairs that are similar and dissimilar clusters to be assigned to the different and the same clusters, respectively. RI is defined as

$$\mathrm{RI} = \frac{TP + TN}{TP + FP + TN + FN} = \frac{TP + TN}{\binom{N}{2}}. \tag{4}$$

RI lies in $[0, 1]$ and high RI means high true positive and true negative rate.

Normalized mutual information (NMI) measures cluster assignment accuracy thorough mutual information entropy,

$$\mathrm{NMI} = \frac{I(\Omega, \mathcal{C})}{H(\Omega)H(\mathcal{C})}, \tag{5}$$

where $I(\Omega, \mathcal{C}) = \sum_k \sum_j \frac{|\omega_k \cap c_j|}{N} log \frac{|\omega_k \cap c_j|}{N}$ and $H(\Omega) = -\sum_k \frac{\omega_k}{N} log \frac{\omega_k}{N}$. NMI lies in $[0, 1]$ and high NMI means that subgroups of objects forms meaningful clusters, *e.g.,* non random cluster assignments.

S-2 Sampling effects on the clustering results

We examined how the clustering results of phyC depend on the sampling effects as follows. We downsampled the number of samples in all patients to be the same (minimum number of samples among patients) and the cancer evolutionary trees based on the downsampled VAF profiles were clustered by phyC. In the down sampling, we randomly chose the subsamples from a patient. To consider the randomness, we took median over the distances of the trees that are obtained from 100 sets of downsampled VAF profiles and the evolutionary trees based on each set of VAF profile.

We made the contingency table where rows and columns represent cluster assignments without downsampling and the assignments with downsampling (Table S4,S5,and S6).

The cluster assignments were the same between those without and with downsampling in ccRCC and NSCLC datasets, respectively (Table S4 amd S5). In case of the dataset of ccRCC&NSCLC, only ccRCC-EV005 was classified in the different clusters compared to the cluster assignments without downsampling (Table S6). There is a long edge in the branch of ccRCC-EV005, and it is considered to be attribute to the cluster assignments without downsampling, compared to those with downsampling.

Our method captures both tree topologies and shapes by edge length vector as shown in equation (2) in the manuscript and this indicates that phyC tends to emphasize the differences of edge length rather than tree topologies, which is implied in the simulation II and III in the manuscript. The number of samples may lead to more complex tree topologies rather than edge length in actual case and it is considered that there was no big difference in clustering results.

Table A: Clustering result of Downsampling of ccRCC

	Cluster 1 (Downsampling)	Cluster 2 (Downsampling)
Cluster 1 (Original)	2	0
Cluster 2 (Original)	0	6

Table B: Clustering result of Downsampling of NSCLC

	Cluster 1(Downsampling)	Cluster 2 (Downsampling)
Cluster 1 (Original)	8	0
Cluster 2 (Original)	0	3

Table C: Clustering result of Downsampling of ccRCC&NSCLC

	Cluster 1 (Downsampling)	Cluster 2 (Downsampling)
Cluster 1 (Original)	10	0
Cluster 2 (Original)	1	8

S-3 Supplementary tables and figures

Table D: Results of simulation I.

	Index	phyC	TED	SPD
simulation 1	PR	0.962 (0.039)	0.607 (0.092)	0.571 (0.071)
(op=2)	NMI	0.947 (0.049)	0.186 (0.029)	0.214 (0.037)
	RI	0.973 (0.026)	0.576 (0.06)	0.622 (0.04)
simulation 2	PR	0.903 (0.064)	0.613 (0.101)	0.567 (0.073)
(op=3)	NMI	0.868 (0.082)	0.188 (0.036)	0.204 (0.038)
	RI	0.931 (0.047)	0.571 (0.069)	0.622 (0.043)
simulation 3	PR	0.882 (0.058)	0.557 (0.081)	0.554 (0.07)
(op=4)	NMI	0.827 (0.073)	0.178 (0.032)	0.197 (0.036)
	RI	0.915 (0.041)	0.614 (0.053)	0.626 (0.044)
simulation 4	PR	0.871 (0.065)	0.518 (0.07)	0.544 (0.062)
(op=5)	NMI	0.813 (0.08)	0.173 (0.035)	0.185 (0.034)
	RI	0.91 (0.042)	0.632 (0.043)	0.624 (0.04)

Table E: Results of simulation II.

	Index	phyC	BScore
simulation 1	PR	0.746 (0.057)	0.713 (0.099)
(s=1)	NMI	0.458 (0.099)	0.167 (0.081)
	RI	0.725 (0.049)	0.527 (0.062)
simulation 2	PR	0.806 (0.064)	0.671 (0.1)
(s=2)	NMI	0.586 (0.111)	0.19 (0.085)
	RI	0.791 (0.057)	0.56 (0.055)
simulation 3	PR	0.836 (0.057)	0.658 (0.097)
(s=3)	NMI	0.645 (0.092)	0.195 (0.088)
	RI	0.82 (0.051)	0.567 (0.054)
simulation 4	PR	0.875 (0.064)	0.659 (0.097)
(s=4)	NMI	0.716 (0.11)	0.207 (0.094)
	RI	0.856 (0.065)	0.576 (0.051)
simulation 5	PR	0.888 (0.064)	0.659 (0.096)
(s=5)	NMI	0.747 (0.121)	0.215 (0.099)
	RI	0.871 (0.065)	0.582 (0.05)
simulation 6	PR	0.895 (0.055)	0.637 (0.084)
(s=6)	NMI	0.758 (0.102)	0.192 (0.104)
	RI	0.877 (0.057)	0.582 (0.05)
simulation 7	PR	0.919 (0.056)	0.662 (0.085)
(s=7)	NMI	0.807 (0.104)	0.222 (0.094)
	RI	0.904 (0.058)	0.59 (0.055)
simulation 8	PR	0.922 (0.053)	0.671 (0.084)
(s=8)	NMI	0.814 (0.108)	0.247 (0.111)
	RI	0.907 (0.058)	0.602 (0.053)
simulation 9	PR	0.936 (0.047)	0.649 (0.093)
(s=9)	NMI	0.845 (0.097)	0.23 (0.111)
	RI	0.922 (0.054)	0.593 (0.05)
simulation 10	PR	0.947 (0.049)	0.666 (0.095)
(s=10)	NMI	0.874 (0.103)	0.235 (0.102)
	RI	0.937 (0.055)	0.595 (0.052)

Table F: Results of simulation III.

	Index	phyC	BScore
simulation 1	PR	0.56 (0.041)	0.724 (0.059)
(s=1)	NMI	0.508 (0.038)	0.42 (0.058)
	RI	0.848 (0.011)	0.671 (0.08)
simulation 2	PR	0.642 (0.046)	0.743 (0.05)
(s=2)	NMI	0.599 (0.04)	0.495 (0.049)
	RI	0.868 (0.012)	0.729 (0.041)
simulation 3	PR	0.68 (0.045)	0.757 (0.058)
(s=3)	NMI	0.643 (0.038)	0.536 (0.064)
	RI	0.878 (0.011)	0.752 (0.047)
simulation 4	PR	0.704 (0.053)	0.769 (0.053)
(s=4)	NMI	0.667 (0.035)	0.566 (0.053)
	RI	0.882 (0.01)	0.771 (0.05)
simulation 5	PR	0.732 (0.053)	0.767 (0.055)
(s=5)	NMI	0.698 (0.037)	0.582 (0.056)
	RI	0.889 (0.013)	0.787 (0.04)
simulation 6	PR	0.736 (0.054)	0.771 (0.055)
(s=6)	NMI	0.701 (0.042)	0.587 (0.058)
	RI	0.89 (0.013)	0.787 (0.041)
simulation 7	PR	0.757 (0.05)	0.774 (0.054)
(s=7)	NMI	0.722 (0.038)	0.615 (0.05)
	RI	0.894 (0.014)	0.81 (0.036)
simulation 8	PR	0.772 (0.046)	0.782 (0.051)
(s=8)	NMI	0.732 (0.037)	0.622 (0.051)
	RI	0.895 (0.015)	0.814 (0.032)
simulation 9	PR	0.777 (0.053)	0.779 (0.058)
(s=9)	NMI	0.738 (0.036)	0.624 (0.059)
	RI	0.895 (0.013)	0.817 (0.033)
simulation 10	PR	0.777 (0.055)	0.769 (0.054)
(s=10)	NMI	0.741 (0.037)	0.626 (0.048)
	RI	0.896 (0.013)	0.823 (0.03)

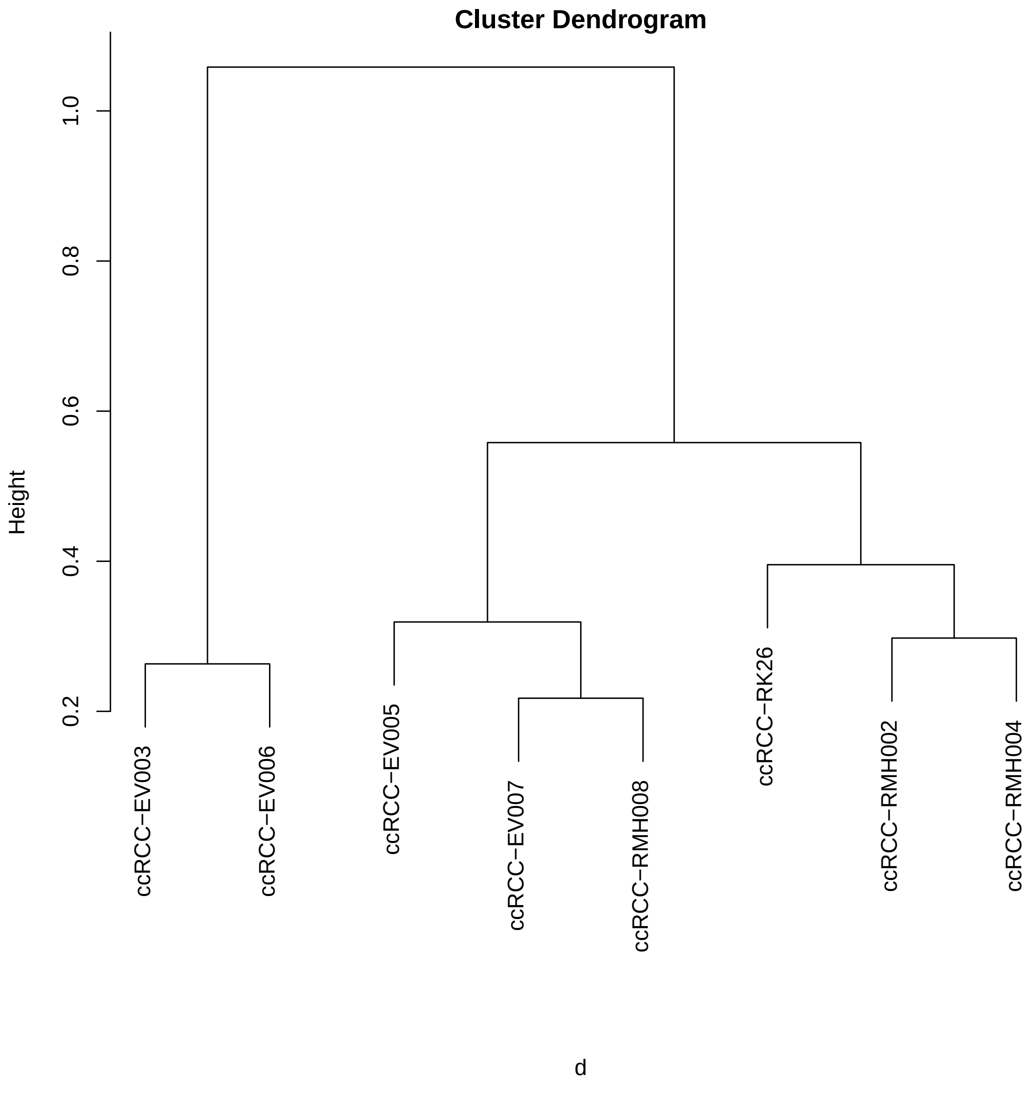

Figure A : Dendrogram of the clustering result of ccRCC data

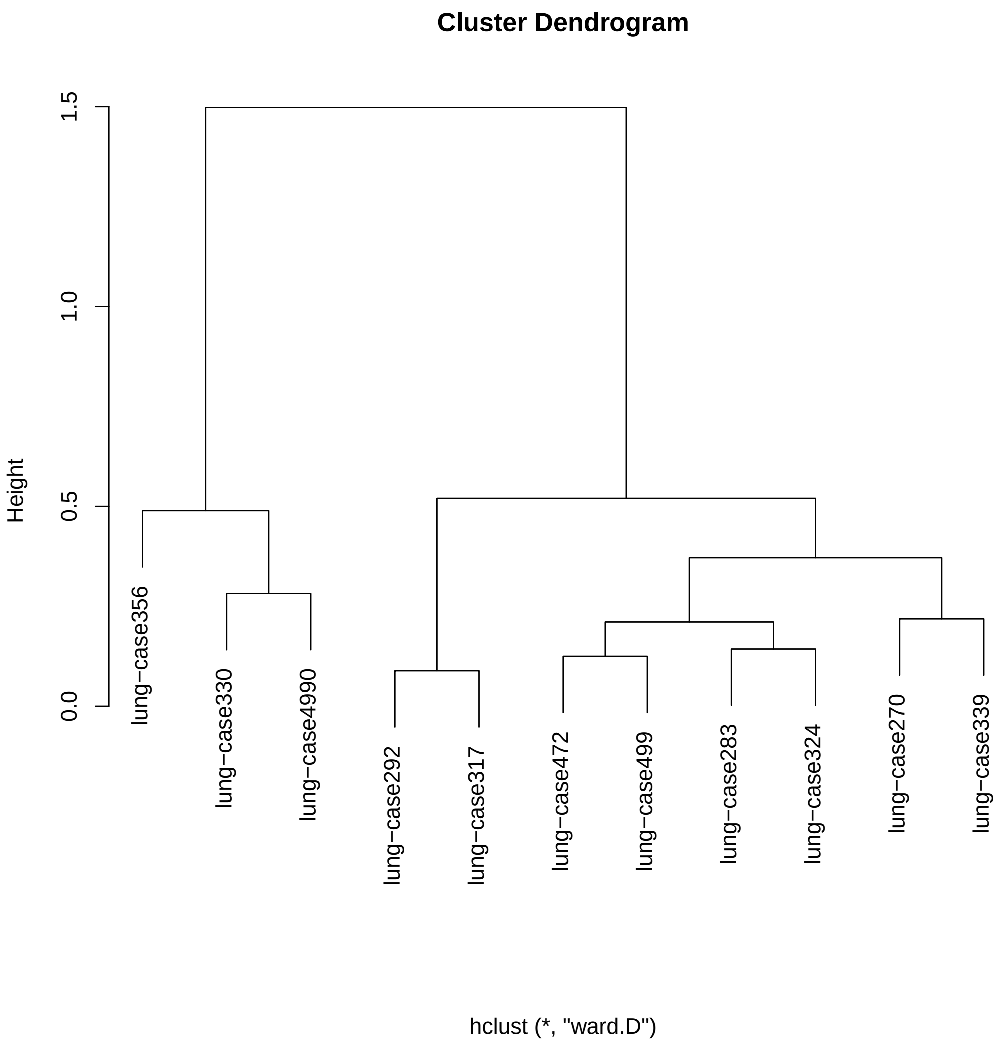

Figure B : Dendrogram of the clustering result of NSCLC data

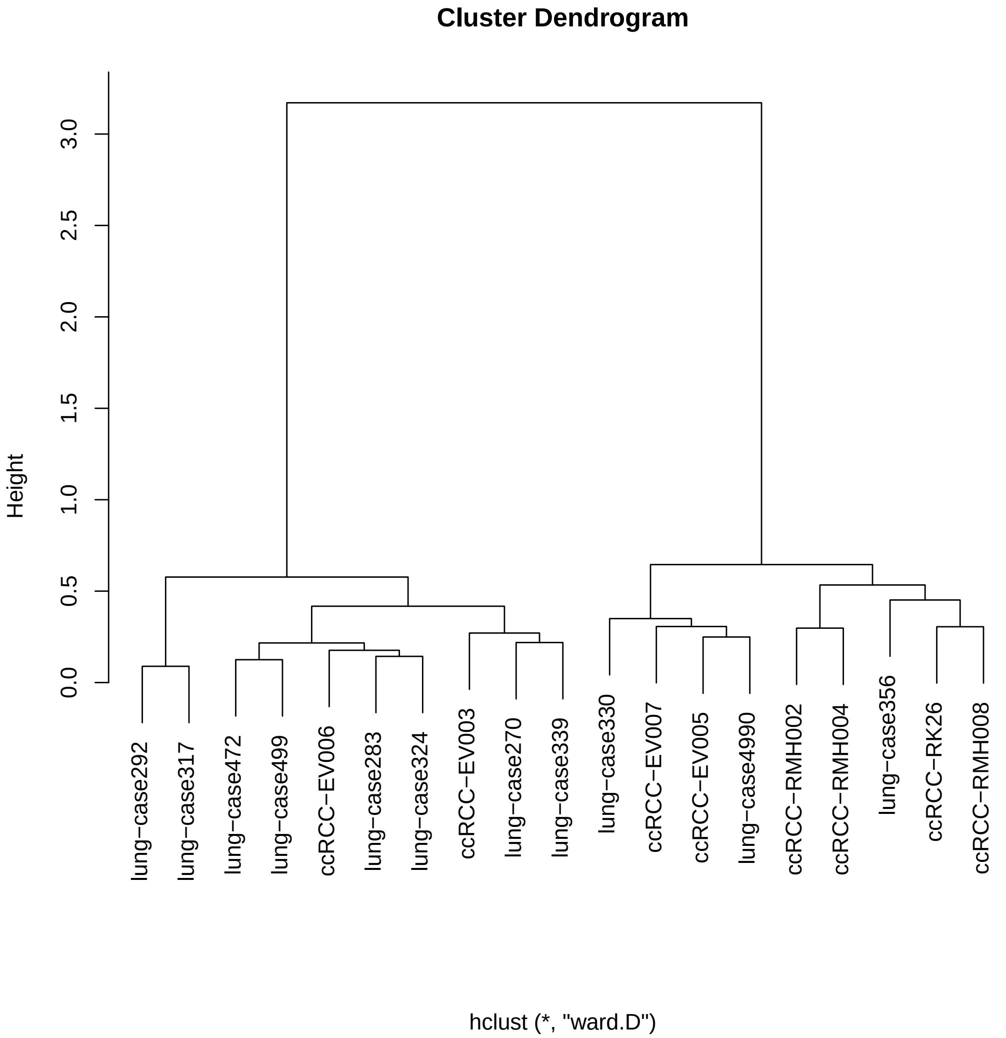

Figure C : Dendrogram of the clustering result of ccRCC&NSCLC data

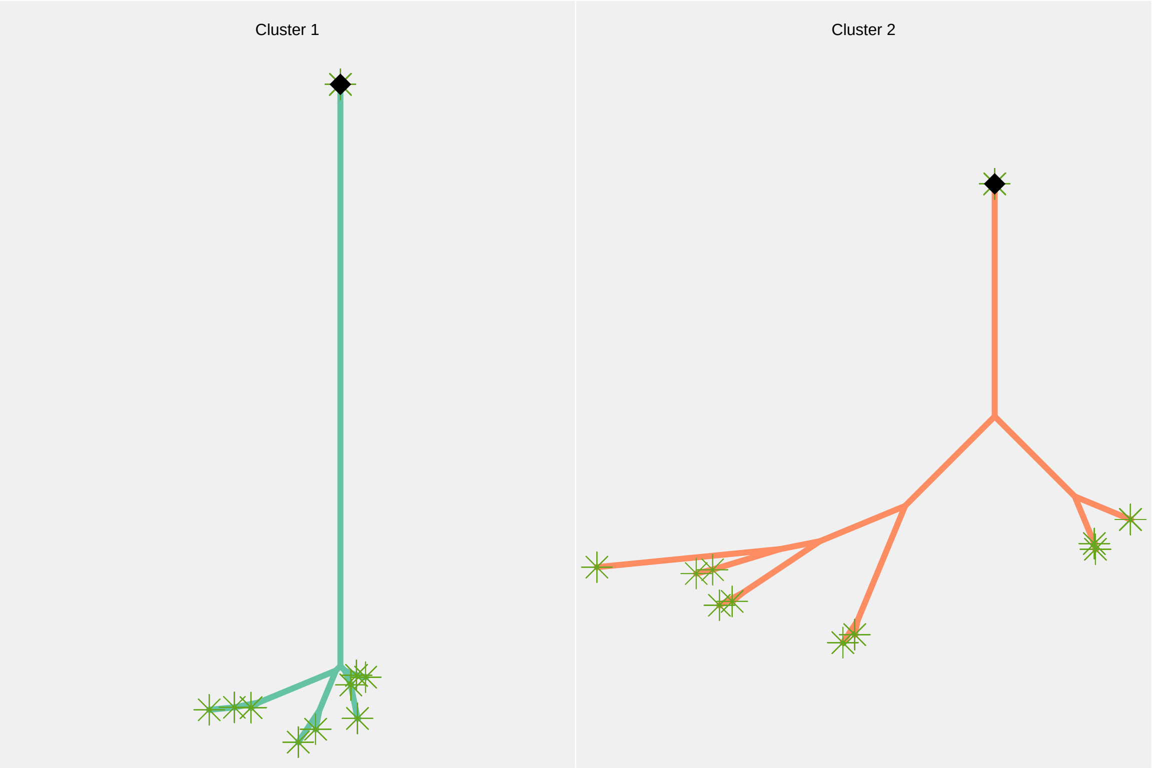

Figure D : Tree averages in the clusters of ccRCC data

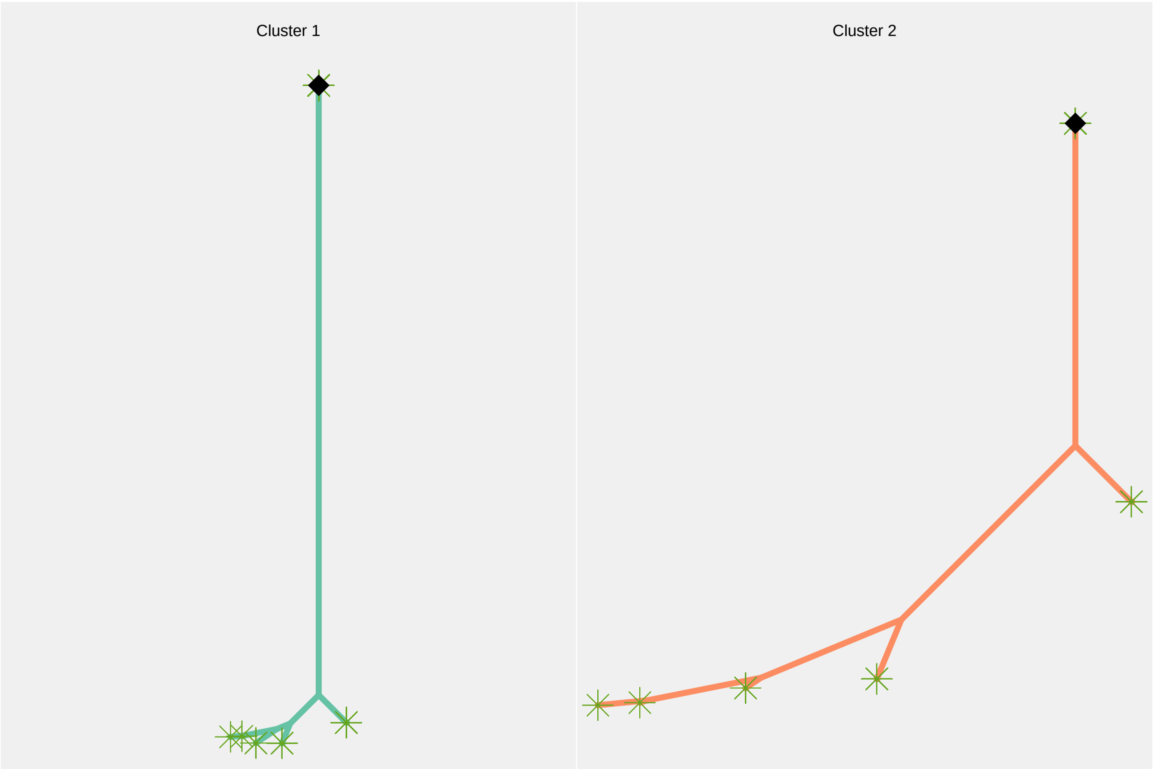

Figure E : Tree averages in the clusters of NSCLC data

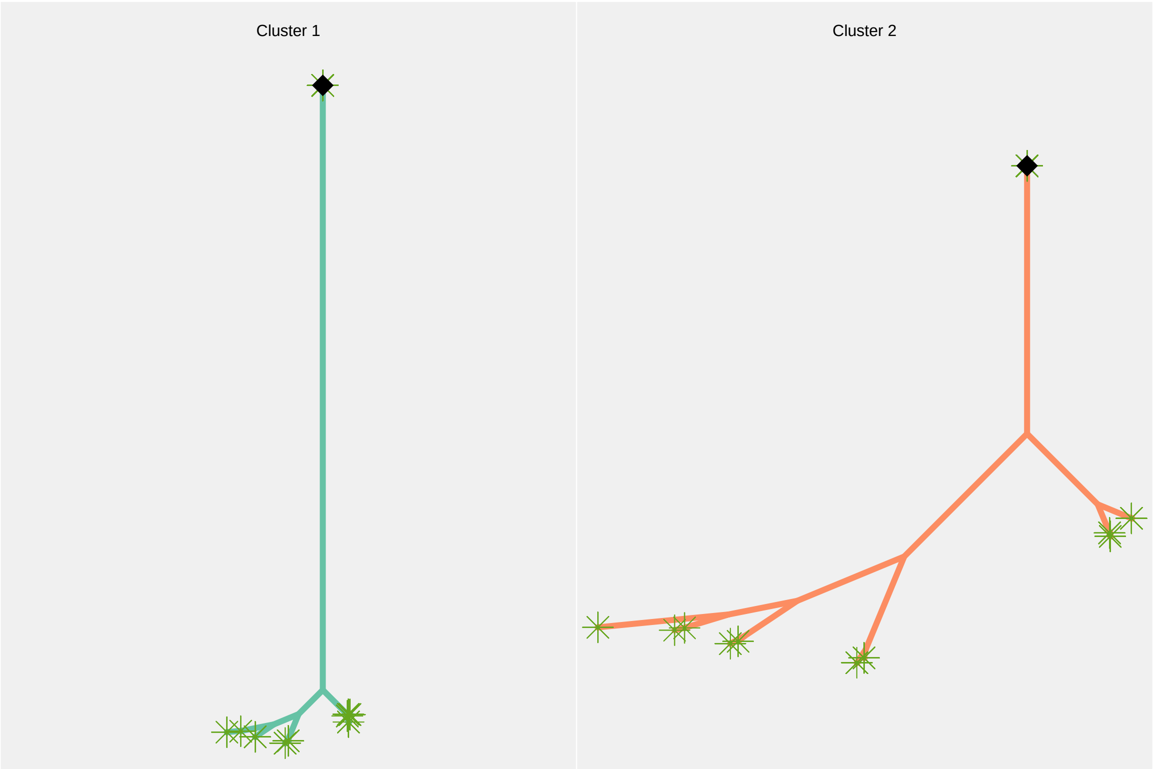

Figure F : Tree averages in the clusters of ccRCC&NSCLC data

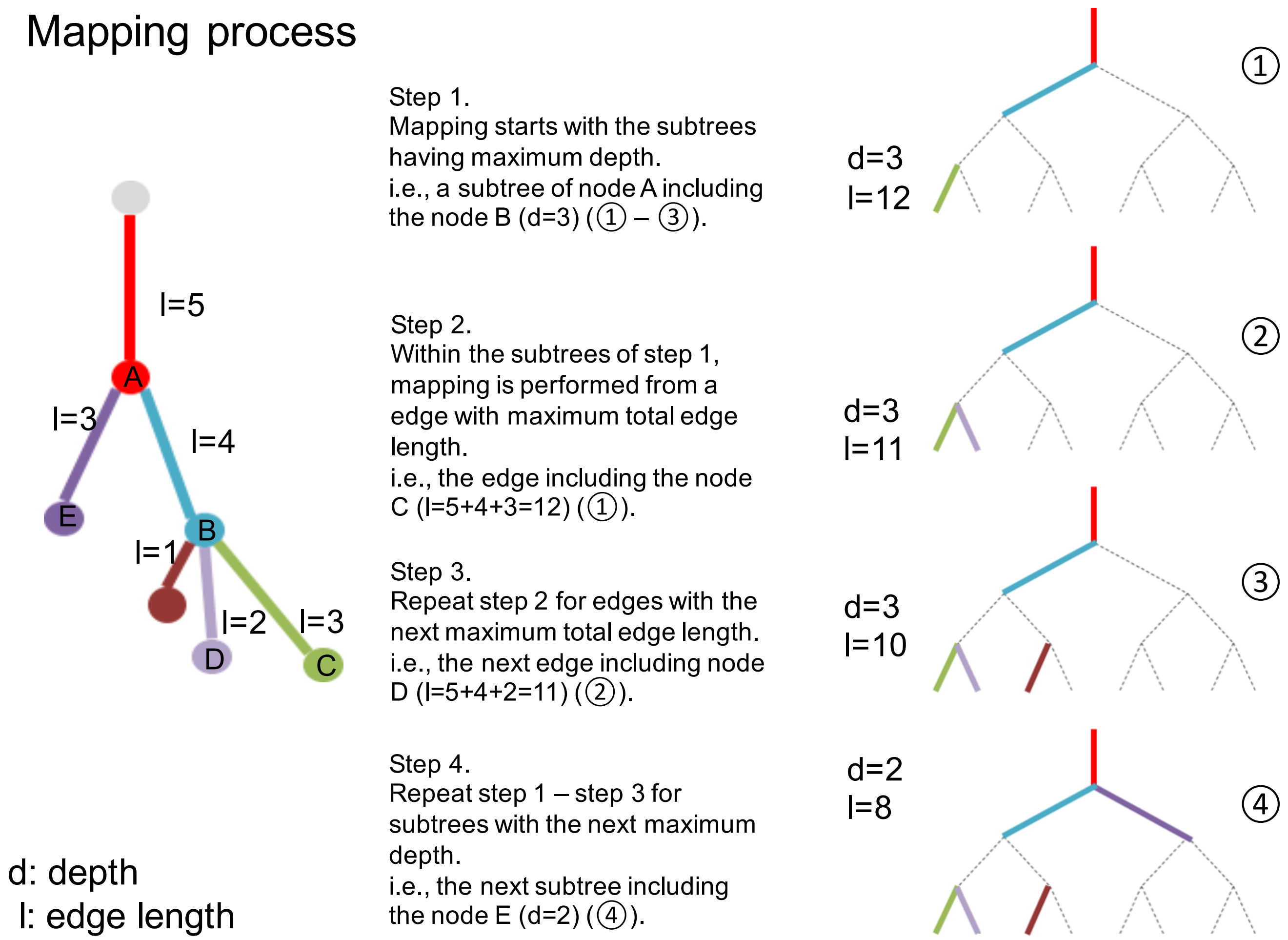

Figure G : Details of mapping process in the registration
