# Supplementary material for: phyC: Clustering cancer evolutionary trees
Source: PLoS Comput Biol. 2017 May 1;13(5):e1005509. doi: 10.1371/journal.pcbi.1005509 (PMC5432190; doi:10.1371/journal.pcbi.1005509)
Supplement: S1 File — (HTML) [file pcbi.1005509.s002.html]

Reproducing figures and tables in phyC: Clustering cancer evolutionary trees


# Reproducing figures and tables in phyC: Clustering cancer evolutionary trees

#### *Yusuke Matsui*

#### *1.3.2017*

# Reproducing Figure 2

```
root <- 1
mc <- make_empty_graph() %>%
  add_vertices(2) %>%
  add_edges(c(root,2)) 

pl <- make_empty_graph() %>%
  add_vertices(8) %>%
  add_edges(c(root,2,2,3,2,4,3,5,3,6,4,7,4,8)) 

pm <- make_empty_graph() %>%
  add_vertices(16) %>% add_edges(c(root,2,2,3,2,4,3,5,3,6,4,7,4,8,5,9,5,10,6,11,6,12,7,13,7,14,8,15,8,16))

ph <- make_empty_graph() %>%
  add_vertices(32) %>% add_edges(c(root,2,2,3,2,4,3,5,3,6,4,7,4,8,5,9,5,10,6,11,6,12,7,13,7,14,8,15,8,16,9,17,9,18,10,19,10,20,11,21,11,22,12,23,12,24,13,25,13,26,14,27,14,28,15,29,15,30,16,31,16,32))

mut <- make_empty_graph() %>%
  add_vertices(8) %>%
  add_edges(c(root,2,root,3,root,4,root,5,root,6,root,7,root,8)) 

treeList <- list(mc,pl,pm,ph,mut)
edgeList <- lapply(treeList,function(x)get.edgelist(x))
edgeLenList <- lapply(edgeList,function(x)rep(1,nrow(x)))
trees_sim <- phyC(edgeList,edgeLenList,cluster = 5)
```

```
## staring to resolve mono and multifurcations 
## creating maximum tree
## depth=6 and #leaves=64
## starting encoding trees to maximum tree
## 1 th registration finished
## 2 th registration finished
## 3 th registration finished
## 4 th registration finished
## 5 th registration finished
## hierarchical clustering
```

```
treeLabel <- c("Mono clonal","Polyclonal low","Polyclonal middle","Polyclonal high","Mutator phenotype")
phyC.plot(trees_sim,nPanel = c(1,5), label = treeLabel)
```

# Reproducing Figure 3

```
load("fig4.RData") # fig4.RData can be downloaded from https://github.com/ymatts/phyC/misc
methodColor <- brewer.pal(4,"Set2")
names(methodColor) <- c("phyC","SPD","TED","BScore")
selectColor <- list(1:3,c(1,4),c(1,4))
methodColorList <- lapply(selectColor,function(x)methodColor[x])

ggList <- vector("list",9)
g <- 1
for(i in 1:3){
  selectDat <- dat[dat$simName==paste0("sim",i),]
  evalName <- unique(as.character(selectDat$evalName))
  for(j in seq_along(evalName)){
    selectDat2 <- selectDat[selectDat$evalName==evalName[j],]
    selectDat2 <- cbind(selectDat2, ymin = pmax(0,selectDat2$Index - 1.96 * selectDat2$SD),ymax = pmin(1,selectDat2$Index  + 1.96 * selectDat2$SD))
    gg <- ggplot(selectDat2,aes(x = varPar,y = Index,color = method))
    gg <- gg + 
      geom_line(size=1.3) + 
      ylim(c(0,1)) + 
      scale_color_manual(values = methodColorList[[i]]) + 
      geom_ribbon(aes(ymin = ymin, ymax = ymax, fill = method, color = NULL),alpha = 0.2) +
      scale_fill_manual(values = methodColorList[[i]])
    print(gg)
  }
}
```

# Reproducing Figure 4 and Figure S1-S6

Note that in MDS configuration, coordinates can be different from in the manuscript although distances between trees are the same.

```
demo <- function(vaf,thr,g=1){
  label <- names(vaf)  
  trees <- vector("list",length(vaf))
  for(i in seq_along(vaf)){
    trees[[i]] <- invisible(phyC::par.tree(vaf[[i]]))
  }
  
  edgeList <- lapply(trees,function(x)x$edge)
  edgeLenList <- lapply(trees,function(x)x$edge.length)
  min.nc <- 2
  max.nc <- 6
  res <- invisible(phyC::phyC(edgeList,edgeLenList,min.nc = min.nc, max.nc = max.nc,type = "h",method = "ward.D"))
  k <- max(unique(res$cluster))
  cmd <- phyC::phyCMD(res,label = label,label.size = 3)
  div <- phyC::diversity(res)
  plot(hclust(res$dist,method="ward.D"),labels = label,xlab = "")
  if(g == 1){
    phyC.plot(res,label = paste("Cluster",1:k),nPanel = c(1,k),ave = T,nodesize=4.5,tipsize=2,edge.width = 3)
  }else if(g == 2){
    phyC.plot(res,label = paste("Cluster",1:k),nPanel = c(1,k),ave = T,nodesize=3.5,tipsize=1.5,edge.width = 3)
  }
}
```

```
thr <- 0.05 # threshold of VAF
```

Generating figure 4; A-1 and B-1, and figure S1

```
data(ccRCC)
vaf <- lapply(ccRCC,function(x)x[,-(1:3)]) # ccRCC
demo(vaf,thr,g = 1)
```

```
## staring to resolve mono and multifurcations 
## creating maximum tree
## depth=7 and #leaves=128
## starting encoding trees to maximum tree
## 1 th registration finished
## 2 th registration finished
## 3 th registration finished
## 4 th registration finished
## 5 th registration finished
## 6 th registration finished
## 7 th registration finished
## 8 th registration finished
## hierarchical clustering
```

Generating figure 4; A-2 and B-2, and figure S2

```
data(NSCLC)
vaf <- lapply(NSCLC,function(x)x[,-(1:3)]) # NSCLC
demo(vaf,thr,g = 2)
```

```
## staring to resolve mono and multifurcations 
## creating maximum tree
## depth=6 and #leaves=64
## starting encoding trees to maximum tree
## 1 th registration finished
## 2 th registration finished
## 3 th registration finished
## 4 th registration finished
## 5 th registration finished
## 6 th registration finished
## 7 th registration finished
## 8 th registration finished
## 9 th registration finished
## 10 th registration finished
## 11 th registration finished
## hierarchical clustering
```

Generating figure 4; A-3 and B-3, and figure S3

```
data(ccRCC_NSCLC)
vaf <- lapply(ccRCC_NSCLC,function(x)x[,-(1:3)]) #ccRCC_NSCLC
demo(vaf,thr,g = 2)
```

```
## staring to resolve mono and multifurcations 
## creating maximum tree
## depth=7 and #leaves=128
## starting encoding trees to maximum tree
## 1 th registration finished
## 2 th registration finished
## 3 th registration finished
## 4 th registration finished
## 5 th registration finished
## 6 th registration finished
## 7 th registration finished
## 8 th registration finished
## 9 th registration finished
## 10 th registration finished
## 11 th registration finished
## 12 th registration finished
## 13 th registration finished
## 14 th registration finished
## 15 th registration finished
## 16 th registration finished
## 17 th registration finished
## 18 th registration finished
## 19 th registration finished
## hierarchical clustering
```
